# Mortality following *Campylobacter *infection: a registry-based linkage study

**DOI:** 10.1186/1471-2334-5-70

**Published:** 2005-09-14

**Authors:** Anders Ternhag, Anna Törner, Åke Svensson, Johan Giesecke, Karl Ekdahl

**Affiliations:** 1 Department of Epidemiology, Swedish Institute for Infectious Disease Control, Solna, Sweden; 2 Department of Medical Epidemiology and Biostatistics, Karolinska Institutet, Stockholm, Sweden; 3 Division of Mathematical Statistics, Stockholm University, Sweden

## Abstract

**Background:**

Campylobacteriosis is one of the most commonly identified causes of bacterial diarrheal disease and a common cause of gastroenteritis in travellers from developed nations. Despite the widespread occurrence, there is little information on *Campylobacter *mortality.

**Methods:**

Mortality among a cohort of *Campylobacter *cases were compared with the general population 0–1, 1–3, 3–12 and more than 12 month after the onset of the illness. The cases were sub-grouped according to if they had been infected domestically or abroad.

**Results:**

The standardized mortality ratio for cases infected domestically was 2.9 (95% CI: 1.9–4.0) within the first month following the illness. The risk then gradually diminished and approached 1.0 after one year or more have passed since the illness. This initial excess risk was not attributable to any particular age group (such as the oldest).

In contrast, for those infected abroad, a lower standardized mortality ratio 0.3 (95% CI: 0.04–0.8) was shown for the first month after diagnosis compared to what would be expected in the general population.

**Conclusion:**

Infection with *Campylobacter *is associated with an increased short-term risk of death among those who were infected domestically. On the contrary, for those infected abroad a lower than expected risk of death was evident. We suggest that the explanation behind this is a "healthy traveler effect" among imported cases, and effects of a more frail than average population among domestic cases.

## Background

Campylobacteriosis is a zoonotic disease observed in most parts of the world. The disease is caused by *Campylobacter jejuni*, or less commonly *Campylobacter coli*. It is estimated to cause 5–14% of diarrhea, worldwide [1], and causes a great amount of hospital bed days in all countries. In the United States, campylobacteriosis account for approximately 17% of hospitalizations due to food borne infections [2], and in England and Wales *Campylobacter *is by far the most important food-borne pathogen when it comes to GP consultations, hospital admissions and hospital bed days [3]. In Sweden, campylobacteriosis is the most common bacterial food-borne disease. Since 1997, between 6,900 and 8,600 annual cases (78–96 per 100,000) have been reported to the Swedish Institute for Infectious Disease Control (SMI), of which approximately 55% were imported [4].

Apart from diarrhea, many patients experience abdominal pain, often accompanied by fever, malaise and headache. The diarrhea can range from mild to profuse, causing dehydration. There are also some known although rare complications to *Campylobacter *infection; Guillain-Barré syndrome is thought to occur after 1 in 1,000 cases of *C*. *jejuni *infection [5,6]. This involves autoantibodies against distinct surface polysaccharides that are similar to host ganglioside structures [7]. Reactive arthritis and Reiter's syndrome (asymmetric arthritis, urethritis and ophthalmitis) are other extra-intestinal manifestations caused by *Campylobacter*, which affects approximately 1% of cases [8].

Although *Campylobacter *infection is both very common and can sometimes result in severe complications, less is known about the mortality attributable to *Campylobacter *infection. In this study we therefore via registry linkage estimated the mortality in a cohort of *Campylobacter *cases in Sweden. The primary objective was to investigate if cases diagnosed with *Campylobacter *have a mortality rate from all causes that differs from the general population. If so, the second objective was to see if this all cause mortality rate among campylobacterosis cases mirrors Campylobacter related mortality rates. We would in this case expect any elevated mortality rate in the Campylobacter cohort to be highest close to infection and then gradually diminish with time and approach the general mortality rate in the population.

## Methods

Sweden has a dual reporting system for notifiable infectious diseases. This means that both the doctor having seen the patient and the laboratory having isolated the pathogen are required to file a report to the SMI. Using a unique personal identification number, issued to all Swedish residents, and used in all contacts with the health care, reports from the two sources are merged into case files.

For this study, we identified all persons reported with campylobacteriosis to the SMI between January 1, 1997 and December 31, 2003. For each case we extracted date of birth, sex, country of infection, date of disease onset, date of diagnosis and reporting date from the database. If the date for disease onset or date of diagnosis was missing we used the median time from patients who have all three dates to back-calculate a date for disease onset. These cases were then via the personal identification number linked to a database that contain information from the National Tax Board on every deceased person in Sweden and their date of death.

We then calculated follow-up time per age group, from the date of disease onset until either an event took place (death) or until November 1, 2004. The following time strata were used; 0–1 month, 1–3 months, 3–12 months and more than 12 months after the onset of campylobacteriosis. The first time strata was constructed to be long enough so that acute effects of even prolonged illness was not missed. The other cut-off was chosen so that effects between strata could be evident (for example a declining risk with time) and that each time strata contained a sufficient amount of person-time for robust estimations. From Statistics Sweden we obtained sex- and age-specific death rates, which were used to obtain the expected number of deaths. Standardized mortality ratios (SMRs) were calculated by dividing the observed number of deaths in our cohort with the expected number of deaths. If the result was >1.0, then the mortality in the *Campylobacter *cohort was higher than in the general population, and conversely, if the ratio was <1.0 then the mortality among our cases was lower than in the general population. The SMR is a weighted average of all age groups in the *Campylobacter *cohort. This indirect standardization was done with 5-year age strata (e.g. 0–4, 5–9, etc.) but is presented here either as SMR, or grouped into broader categories e.g. 0–14 years, 14–64 years and +65 years.

With the assumption that those who had acquired their infection abroad and those who had contracted it at home were two fundamentally different groups – the former probably healthier than the latter ("healthy traveler effect") – we divided our *Campylobacter *cohort into two groups, imported and domestic cases [9].

Exact confidence intervals and tests were calculated assuming that the number of deaths in each stratum was Poisson distributed. Within each time stratum the observed SMRs for each age group were compared using an exact test. The observed number of cases was assumed to be multinomially distributed over the age classes with probabilities proportional to the expected values. All calculations were performed using SAS statistical software, V. 8.2.

## Results

During the study period 1997–2003, 48,025 unique individuals were notified with *Campylobacter *infection, of whom 16,710 were infected in Sweden, and 28,930 were infected abroad. For the remaining 2,385 cases either the personal identification number was incomplete, or the country of infection was unknown.

For cases reported with *Campylobacter *and infected in Sweden, the SMR was higher compared to the general Swedish population (Table 1). The risk was highest within the first month after onset of disease (SMR 2.9; 95% CI 1.9–4.0). After that time period the risks gradually decreased and after one year had passed after the debut of illness, the risk was the same as for the general population (SMR 1.0; 95% CI 0.9–1.1)

**Table 1:** Standardized mortality ratios (SMR) during the period 1997–2003 among 45,640 Swedish reported cases of campylobacteriosis, infected in Sweden (n = 16,710) and abroad (n = 28,930)

	Infected in Sweden			Infected abroad		
		
Time after infection (months)	Obs* (n = 563)	Exp*	SMR* (95% CI*)	Obs (n = 222)	Exp	SMR (95% CI)
0–1	30	10.4	2.9 (1.9–4.0)	2	6.8	0.3 (0.04–0.8)
1–3	31	20.7	1.5 (1.0–2.1)	10	13.7	0.7 (0.4–1.3)
3–12	123	93.9	1.3 (1.1–1.6)	28	63.3	0.4 (0.3–0.6)
>12	379	388.6	1.0 (0.9–1.1)	182	330.6	0.6 (0.5–0.6)

* Obs, observed number of deaths; Exp, expected number of deaths; SMR, standardized mortality ratio; CI, confidence interval

To see if this early excess mortality risk was attributable to any particular age group within the group of domestically infected individuals, we divided the cohort into three age groups (0–14 years, 15–64 years and 65 years and above). Table 2 shows the mortality ratios for these separate age groups. No significant differences between the groups were found at any of the time periods following the illness (the lowest p-value is 0.20). Persons infected with *Campylobacter *abroad, had lower SMR compared to the general population (Table 1). We observed less number of deaths than expected in every time stratum after the debut of illness. Within the first month after the disease SMR was 0.3 (95% CI 0.04–0.8), and a significantly lower risk compared to the reference population was evident for every time period except for the second time stratum (1–3 months after infection) where the SMR was 0.7 (95% CI: 0.4–1.3).

**Table 2:** Standardized mortality ratios (SMR) in age groups 0–14, 15–64 and 65+ years among 16,710 domestically infected Campylobacter cases in Sweden 1997–2003

Time after infection	Age group (years)	Obs*	Exp*	SMR* (95% CI*)
0–1 months	0–14	1	0.1	8.9 (0.2–32.9)
	15–64	4	2.0	2.0 (0.5–4.3)
	65+	25	8.3	3.0 (2.0–4.3)
1–3 months	0–14	0	0.2	0 (0–13.5)
	15–64	7	4.1	1.7 (0.7–3.2)
	65+	24	16.4	1.5 (0.9–2.1)
3–12 months	0–14	3	1.0	3.1 (0.6–7.6)
	15–64	26	18.5	1.4 (0.9–2.0)
	65+	94	74.4	1.3 (1.0–1.5)
>12 months	0–14	0	2.3	0 (0–1.3)
	15–64	72	75.5	1.0 (0.7–1.2)
	65+	307	310.7	1.0 (0.9–1.1)

* Obs, observed number of deaths; Exp, expected number of deaths; SMR, standardized mortality ratio; CI, confidence interval

## Discussion

The SMR was almost three times higher for persons infected with *Campylobacter *in Sweden within the first months after disease onset. These risks diminished with time, and after a year or more have passed the SMR almost equaled that for the general population. For the imported cases of *Campylobacter *we observed lower SMRs throughout every time point in the study period.

Case fatality rate (CFR) is widely used and perhaps more intuitive than standardized mortality ratio when disease specific mortality is estimated. Up until November 1, 2004, 563 of the cases infected in Sweden, and 222 of the cases infected abroad had died. The CFR within 30 days was 0.19% (95% CI 0.13–0.27%) for those infected in Sweden, and 0.008% (95% CI 0.0008–0.03%) for those infected abroad. However, we do not favour the use of CFR because information on competing causes of mortality is missing, along with age effects.

The different SMRs depending on whether a case was infected at home or abroad must be analyzed in a context of how a case is diagnosed and reported. For gastrointestinal diseases there are problems with under-reporting and biases regarding which cases that eventually turns up in the national routine surveillance.

The problems with under-reporting affect every step in the chain: an ill person must seek health care, the doctor needs to take a stool sample, the laboratory must be capable of accurately detecting the pathogen, and if the laboratory find the pathogen the doctor needs to send in a notification. It is perhaps in the first of these steps that national surveillance misses most of the cases. Studies in both United Kingdom and United States have tried to measure the proportion reported to national surveillance from those who have symptoms of infectious intestinal disease in the population. For an agent like *Campylobacter *that typically causes non-bloody diarrhea a multiplier in the range of 7.6–38 has been estimated [10,11].

All persons who get infected with *Campylobacter *do not have the same chance of becoming notified as a *Campylobacter *case. There are three different groups that are more likely than others to be reported [12]. Firstly, people who have a severe disease or profound symptoms are more likely to see a doctor, compared to others. Secondly, persons with a history of recent travel prior to the onset of symptoms are also more likely to seek health care and become diagnosed and reported. Thirdly, persons with pre-existing illnesses of certain magnitude may be over-represented in the national surveillance statistics.

These biases in disease reporting will have an impact on our findings and can help us in the interpretation of the results. The general under-reporting will affect milder cases in particular. Therefore, the persons in our cohort infected in Sweden were most likely the more severe cases in the population. On the other hand, travellers who develop symptoms of gastro-enteritis are more likely on the average to have a physical exam when they have returned home and they do not necessary need to have a severe disease. The finding of a standardized mortality ratio equal to 1.0 after one year had passed after the infection suggest that the mortality of this cohort infected domestically is comparable to the standard Swedish population after one year. This does not necessarily imply that the cohort is comparable to the Swedish population also at the earlier time points. The risk profile may have changed due to frailty. The statistical term frailty implies that all individuals do not have the same risk profile and frailer individuals may succumb earlier and therefore the risk profile of a cohort changes over time [13].

It is difficult to find information on *Campylobacter *associated mortality. Some have estimated the case fatality rate to be in range of 1–3/10000 cases [14]. Others that have used an approach more like ours found that 1.2% (16180 cases and 190 deaths) where deceased within one year from diagnosis [15]. Unfortunately, no study has compared mortality risks among travellers and non-travellers for campylobacteriosis.

Our findings suggest that the individuals reported into the surveillance system are a highly selected. Individuals with a domestically contacted infection may have a different, more severe risk profile, compared to individuals in the cohort with an imported infection. The reason for these differences is not characteristics of the infection itself but reflects differences in how these individuals are selected into the surveillance system. Any registry-based estimation of mortality among cases of campylobacteriosis must therefore take this into account and stratify cases according to the place of infection.

## Conclusion

In conclusion, we found in our study cohort two groups of persons with opposite mortality risks within the first month after a *Campylobacter *infection. Persons who had acquired their infection in Sweden had almost a three times higher than expected risk to die. The larger group of persons, having picked up their infection abroad, had, on the contrary, a lower than expected risk to die, likely due to a "healthy traveler effect". In estimations of mortality or burden of disease regarding campylobacteriosis one should be aware that travellers differ in many characteristics compared to non-travellers. In countries where imported cases are in majority, these two groups must be handled separately to avoid any misinterpretation of the true burden of disease.

## Competing interests

The author(s) declare that they have no competing interests.

## Authors' contributions

ATe assembled and analyzed the data and drafted the manuscript. ATo performed the statistical analyses and revised the article. AS contributed to statistical analysis and critically revised the article. KE and JG participated in the study design and critically revised the article. All authors read and approved the final manuscript.

## Pre-publication history

The pre-publication history for this paper can be accessed here:


